# Systematic characterization of human response to H1N1 influenza vaccination through the construction and integration of personalized transcriptome response profiles

**DOI:** 10.1038/s41598-021-99870-0

**Published:** 2021-10-21

**Authors:** Carlo De Intinis, Margherita Bodini, Denise Maffione, Laurane De Mot, Margherita Coccia, Duccio Medini, Emilio Siena

**Affiliations:** 1 University of Turin, 10124 Turin, Italy; 2 GSK, 53100 Siena, Italy; 3 GSK, 1330 Rixensart, Belgium; 4 Present Address: AizoOn, 10146 Turin, Italy; 5 Present Address: Clarivate Analytics, 2600 Berchem, Belgium; 6 Present Address: Toscana Life Sciences, 53100 Siena, Italy

**Keywords:** Computational biology and bioinformatics, Immunology, Systems biology

## Abstract

Gene expression data is commonly used in vaccine studies to characterize differences between treatment groups or sampling time points. Group-wise comparisons of the transcriptional perturbations induced by vaccination have been applied extensively for investigating the mechanisms of action of vaccines. Such approaches, however, may not be sensitive enough for detecting changes occurring within a minority of the population under investigation or in single individuals. In this study, we developed a data analysis framework to characterize individual subject response profiles in the context of repeated measure experiments, which are typical of vaccine mode of action studies. Following the definition of the methodology, this was applied to the analysis of human transcriptome responses induced by vaccination with a subunit influenza vaccine. Results highlighted a substantial heterogeneity in how different subjects respond to vaccination. Moreover, the extent of transcriptional modulation experienced by each individual subject was found to be associated with the magnitude of vaccine-specific functional antibody response, pointing to a mechanistic link between genes involved in protein production and innate antiviral response. Overall, we propose that the improved characterization of the intersubject heterogeneity, enabled by our approach, can help driving the improvement and optimization of current and next-generation vaccines.

## Introduction

Seasonal influenza is a human respiratory disease, caused by influenza virus infection, which severity ranges from mild symptoms to hospitalization and death^1–3^. To date, annual vaccination represents the most effective intervention for containment of this disease^4,5^.

To foster the development of new and more effective influenza vaccines, several systems biology studies tried to characterize the mechanisms of immunogenicity of these vaccines by searching for biomarkers of both humoral and cellular immune responses^6–10^. Despite the general success in identifying molecular signatures of increased vaccine immunogenicity, these studies highlighted the fact that establishing causality in the chain of reactions triggered by vaccination is not trivial. This is due, at least in part, to the pervasive heterogeneity that characterizes the human population, which results in distinct individuals to respond differently to the same vaccine^11^. Such heterogeneity is likely the result of both heritable (genetic) and non-heritable factors (microbiome, age, diet, etc.), all of which can have a remarkable impact on the immune response to vaccination^12^. A further layer of complexity is introduced by the fact that the immune system is a complex apparatus, with different cells, tissues and organs dynamically interacting with each other over time^13^. This further impairs our ability to understand which immune components should be targeted by a vaccine in order to achieve a broadly protective response.

In contrast to the pervasive heterogeneity characterizing the immune response among different individuals, conventional approaches to the analysis of immunological data are based on group-wise comparisons between groups of individuals (treatment vs control, treatment “A” vs treatment “B”, etc.) and on the resulting identification of signals that are consistently found in most individuals within a study group. Student’s t-test and analysis of variance are two examples of approaches falling in this category. While these represent powerful tools for assessing differences at a group level, they are not suited for capturing subtle signals in noisy datasets or changes occurring in only a small fraction of the study population. For this reason, there have been attempts to explore alternative approaches for characterizing the heterogeneity of biological systems. Among these, Menche *et al.*^14^ developed an approach to derive personalized transcriptome response profiles in individuals affected by either asthma, Parkinson’s or Huntington’s disease. Their method allowed for the characterization of genes being modulated within individual subjects, based on the comparison with a healthy control group. Their approach proved to be more sensitive than conventional techniques in identifying transcriptional signatures associated to a particular disease and allowed to diagnose subjects affected by Huntington’s disease with 100% accuracy.

Inspired by the work of Menche *et al.*, we developed a new data analysis pipeline, for the characterization of individual subject response profiles, and applied it to investigate the transcriptional responses induced by the administration of a subunit influenza vaccine. Our approach identifies genes’ modulation within individual subjects based on the comparison with pre-immune levels. The framework also includes a bootstrap step for estimating the level of confidence in the calls for gene modulation.

Our findings highlighted a high degree of heterogeneity in the transcriptional response to influenza vaccine, both in terms of total number of modulated genes within each individual and in the consistency of modulation of a same gene across multiple study subjects. Despite this, however, genes involved in some key immune related functions showed a more robust response across the study population. Finally, we found the extent of transcriptional perturbation experienced by individual subjects to be correlated with functional antibody response induced by vaccination, with genes involved in protein synthesis and antiviral response driving this association.

Overall, the present study establishes a new approach to the analysis and interpretation of vaccine response data, representing a promising strategy for improving our understanding of the human heterogeneity in vaccine responsiveness.

## Results

### Whole blood transcriptome response induced by influenza vaccination is highly heterogeneous

**Figure 1:**
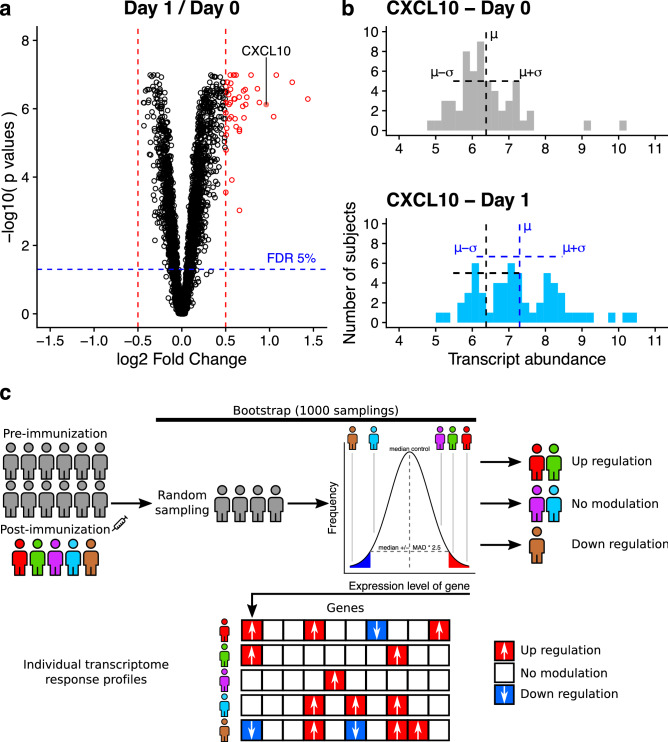
Analytical framework for deriving individual transcriptome response profiles. (**a**) Group-wise DEGs analysis executed on day 1 / day 0 gene expression values (detected DEGs are shown in red). The dashed lines show the cutoffs for calling a gene differentially expressed: log$_{2}$ fold-change $\pm 0.5$ (red lines), adjusted *p*-value $< 0.05$ (two-tailed, paired Wilcoxon signed-rank test, blue line). (**b**) Expression levels distribution for the *CXCL10* gene. While the group-wise comparison suggests a global up-regulation (log$_{2}$ fold-change = 0.96, adjusted *p*-value $= 7.5 \times 10^{-7}$, two-tailed Wilcoxon signed-rank test), some individuals exhibit either absence of modulation or down-regulation. (**c**) Illustration of the proposed approach to derive individual transcriptome response profiles. Instead of comparing case and control groups, each subject is tested individually against the distribution of the gene expression values from the control group. Genes are tested individually and when the expression level is different enough from the control distribution, they are defined as modulated. For each subject an individual transcriptome response profile is generated.

We set out to analyze the transcriptional response to vaccination using publicly available data (Tsang *et al.* 2014) consisting in PBMC transcriptome responses derived from 63 healthy adults vaccinated with the seasonal and pandemic H1N1 subunit influenza vaccines. Conventional, group-wise comparisons identified a set of genes which were significantly upregulated at either 24 hours or 7 days following vaccination (Fig. 1a, Supplementary Fig. S1). Forty-three (43) and 14 differentially expressed genes (DEGs; absolute log$_{2}$ fold-change from baseline $\ge 0.5$, adjusted *p*-value $\le 0.05$, two-tailed Wilcoxon signed-rank test) were identified at 1- and 7-days post-vaccination, respectively. A deeper assessment of transcripts abundance levels, however, revealed a substantial level of heterogeneity across different individuals. Despite the significant modulation observed at the group level, a substantial portion of the study population showed post-vaccination transcript abundance levels comparable (average $\pm 1$ standard deviation) with preimmune levels (Supplementary Table S1 and S2, Supplementary Figure S2).

For exemplary purposes, we report the response levels for the gene encoding for CXCL10, a chemoattractant chemokine involved in the recruitment of T lymphocytes into sites of tissue inflammation^15^. Multiple vaccine studies have reported *CXCL10* to be strongly upregulated in peripheral blood few hours after vaccination, with peak responses typically occurring after 24 h^16^. Consistently with that, we also found the *CXCL10*-encoding gene to be amongst the most strongly upregulated transcripts at the 24 hours’ time point (Fig. 1a; log$_{2}$ fold-change $= 0.96$, adjusted *p*-value $=7.5 \times 10^{-7}$, two-tailed Wilcoxon signed-rank test). Despite the significant *CXCL10* upregulation observed at the group level, this response was not consistently found across all vaccinated subjects. Half of the study subjects (30 out of 60) showed a *CXCL10* transcript abundance comparable (average $\mu \pm 1$ standard deviation $\sigma$) to preimmune levels (Fig. 1b). Moreover, for 5 subjects the *CXCL10* baseline abundance was found to exceed the average level measured 24 hours after vaccination, highlighting a considerable level of intersubject heterogeneity in the response of this transcript. Overall, results indicate that the peripheral blood transcriptome response to influenza vaccination is characterized by a pervasive intersubject heterogeneity.

### Only 55% of day 1 differentially expresses genes are also found within individual subject response profiles

To quantify the intersubject variation in PBMC transcriptome response, we computed individual subject response profiles. Briefly, each subject was tested individually against the distribution of the gene expression values from the control group. Genes were tested individually and when their expression level was different enough from the control distribution, they were defined as modulated. A bootstrap approach was used to limit the effect of outlying values and to assess the robustness of the response (Fig. 1c and methods). After computing the individual subject response profiles, we compared them to the list of DEGs identified through the Wilcoxon test-based group-wise analysis. Despite a substantial overlap, not all DEGs were confirmed by the individual profiles (Supplementary Fig. S3). Among the 43 DEGs observed at day 1, an average of 55% [interquartile range (IQR): 37%-77%] was also captured by the individual profiles, while for day 7 (14 DEGs) the average overlap was 71% (IQR: 42%-100%). This suggests that the average responses captured by group wise analyses do not necessarily represent the behavior of each individual subject. For the day -7 and day 70 time points, which are unlikely to reflect any substantial vaccination-specific effect, the overlap was smaller [day -7 (11 DEGs): 23% (IQR: 0-36%); day 70 (2 DEGs): 20% (IQR: 0-38%); Supplementary Fig. S3]. This is suggestive of the fact that the overlaps observed for day 1 and day 7 are reflective of a real biological perturbation induced by the vaccine stimulus.

**Figure 2:**
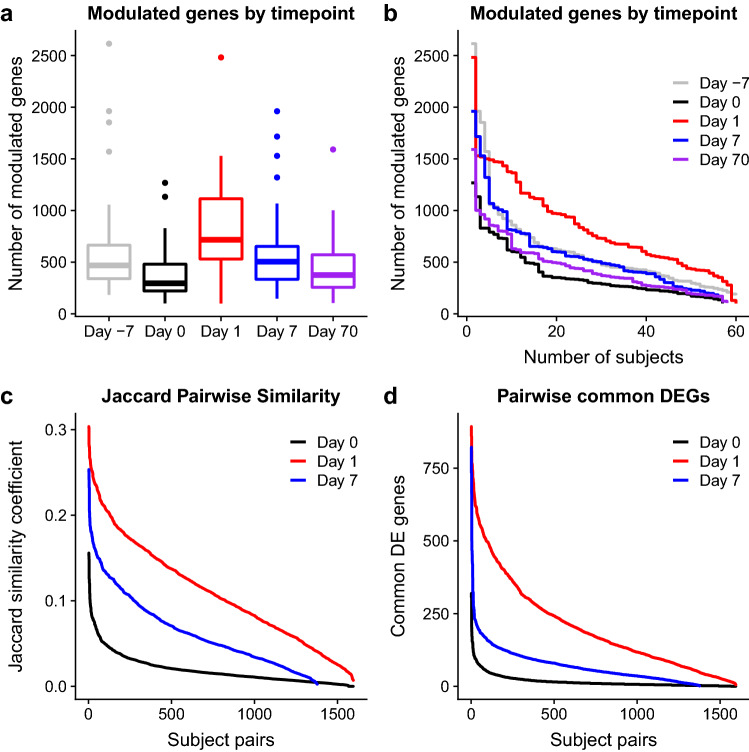
Heterogeneity among individual transcriptome response profiles. (**a**) Number of genes modulated among subjects detected by our analysis. (**b**) Reverse cumulative distributions of number of modulated genes across subjects. (**c**) Jaccard similarity coefficients of pairwise subjects combinations calculated using individual subject transcriptome profiles. (**d**) Number of shared differentially expressed genes (DEGs) among pairwise subjects combinations.

Individual response profiles from day 1 showed the highest number of modulated genes, with an average of 717 genes. The first and third quartiles of that distribution were respectively 531 and 1114 (Fig. 2a,b), reflecting the different response magnitude experienced by the different study subjects. Individual profiles from all other time points showed less modulation, albeit the spread between the 1st and 3rd quartiles remained approximately two-fold (IQR day 7: 334-653; IQR day -7: 340-664; IQR day 70: 256-571). This same trend was also observed with the group-wise differential gene expression analysis (Supplementary Fig. S1).

An additional information gathered from individual transcriptome response profiles was the proportion of individuals in which each gene was found to be modulated. Surprisingly, no gene was found to be consistently modulated across the entire study population, regardless of sampling time point. As we have shown, the most robust response was observed at day 1. At this time point the number of genes found to be modulated in 20 or more subjects, for example, was 587, compared to 84, 120 and 8 measured at days -7, 7 and 70, respectively. We also tested, for days 0, 1 and 7, the pairwise similarity between individual profiles by computing the Jaccard similarity metric and the number of commonly modulated genes (Fig. 2c,d). Both approaches confirmed that the day 1 response was the most consistent. In line with previous observations, however, the generally low similarity coefficients highlight substantial heterogeneity across individual profiles.

Overall, we showed that the individual transcriptome response profiles, generated through our approach, were able to capture vaccination-specific responses while also providing additional information about the consistency of such responses. Specifically, transcriptome responses were found to differ greatly across different subjects and no transcript was found to be consistently modulated across the entire study population.

### Genes participating to immune functions show a more consistent modulation

Next, we tested whether the observed heterogeneity in the transcriptome response is a ubiquitous characteristic or if there are specific biological functions showing a more consistent modulation. To this end, we functionally characterized each individual transcriptome response profile by performing a pathway enrichment analysis. Briefly, we collected a list of canonical pathways from the MSigDB database and tested, for each individual profile, whether the number of modulated genes within a given pathway was higher than random expectation. Through this approach, we could derive a list of enriched pathways from each subject. The level of conservation of such pathways could then be assessed as the proportion of subjects in which they were found to be enriched. A comprehensive listing of the results observed throughout the different time points is presented in Supplementary Tables S3-7.

**Figure 3:**
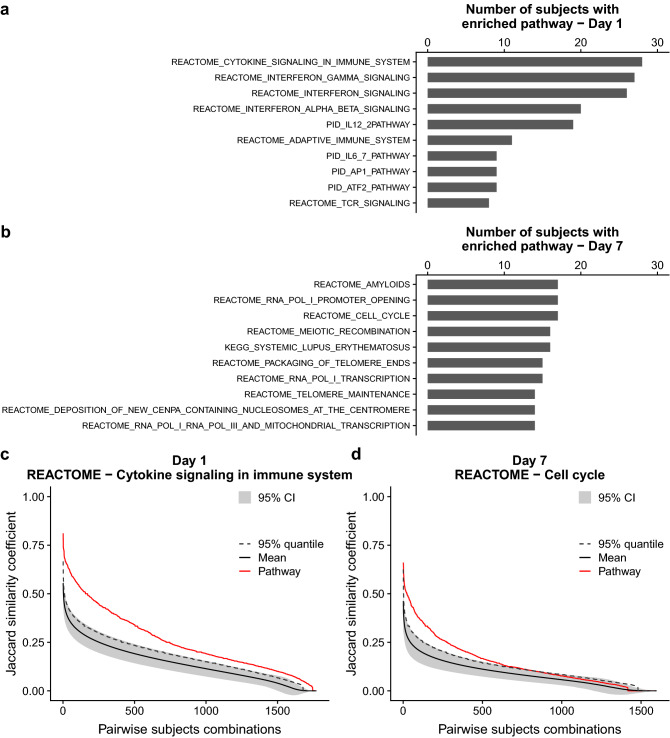
Functional characterization of individual subject response profiles. (**a,b**) Top ten immunological pathways that show enrichment in the highest number of subjects at days 1 and 7, respectively. X-axis: number of subjects in which a pathway is significantly enriched. (**c,d**) Most frequently enriched pathways at days 1 and 7 respectively. Jaccard similarity coefficients represent the robustness of gene modulation for those genes belonging to a specific pathway (red line). For comparison, the robustness of gene modulation of non-in-pathway, randomly selected genes (1000 bootstrap samplings) is also shown. Solid and dashed black lines represent the median and 95th percentile of the bootstrapped distributions. Gray shaded areas represent the 95% confidence interval.

Figure 3a,b lists the 10 most frequently enriched pathways across the study population for days 1 and 7, respectively. Consistently with previous observations from group-wise analyses^6,9,10^, 24 hours transcriptomes showed enrichment for genes involved in cytokine signaling and interferon-mediated antiviral response. Conversely, day 7 responses were enriched for protein transcription and cell proliferation functions, a pattern that is usually associated with B lymphocyte proliferation and differentiation into antibody-producing cells. Similarly to what was observed with individual genes, also the pathways activation was variable across subjects, with the most frequently enriched pathway at 24 h, *cytokine signaling in immune system*, being enriched in less than 50% of the study population (28 out of 60 subjects; Fig. 3a). Seven days post vaccination the response was generally less consistent and pathways were found to be enriched in up to 17 out of 57 subjects (less than 30% of the study population; Fig. 3b).

To test whether the observed functional enrichment was indicative of a true biological signal, the Jaccard pairwise similarity for modulated genes observed in each enriched functional pathway was compared to that of randomly selected gene pools. Genes belonging to the same functional pathway typically showed more robust modulation than randomly assorted genes (Fig. 3c-d and Supplementary Fig. S5-6). Overall, these results are indicative of the fact that, despite the substantial response heterogeneity across different subjects, there is a convergence towards more consistent modulation of genes participating to biological processes involved in the response to influenza vaccination.

### Assessment of vaccination-induced transcriptional modules shows that the intersubject response heterogeneity increases as a function of time

Given the relatively high number of genes within the individual response profiles, there is the chance that genes may appear as modulated across different subjects simply by chance. In the absence of a biological driver, it could be assumed a model of complete independence between subjects and expect the occurrence of perturbations across multiple subjects to follow a random expectation. Evidences collected so far, however, indicate that the vaccination stimulus induces transcriptional perturbations pointing towards common functional disruptions and that a vaccine-induced gene module may exist. In order to characterize such transcriptional module, we applied a combinatorial model to compute the random probability of a modulated gene to appear in multiple subjects and compared it to the available experimental data.

**Figure 4:**
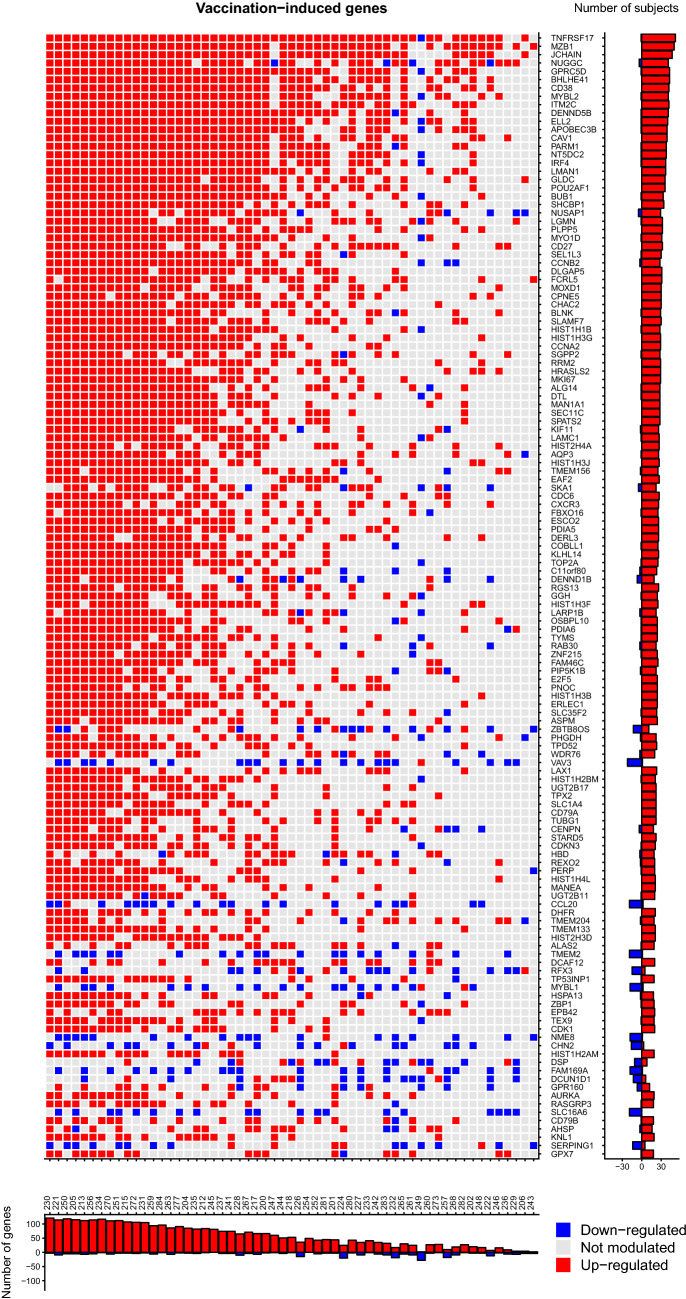
Individual transcriptome response profiles for the day 7 vaccination-induced gene pool. Each row corresponds to a gene and each column to a subject. On the right: number of gene modulations observed across all subjects. On the bottom: number of modulated genes in each study subject.

The combinatorial model indicated that genes co-modulated in $\ge$ 25 and $\ge$ 19 subjects at days 1 and 7, respectively, are unlikely to appear randomly and could be reasonably attributed to a vaccine-related effect. This estimate produced a core-pool of 341 and 135 genes for day 1 and 7 responses, respectively (Fig. 4, Fig. S7 and Supplementary Data 1 and 2). From a functional perspective, the core pool of day 1 modulated genes included an abundance of genes participating in cytokine signaling, mainly related to type-I/II interferon response. The day 7 core pool, instead, was characterized by genes participating in mitotic cell division, likely reflecting B-cell proliferation and differentiation into plasmablasts (supplementary Data 3). Overall, the observed functional profiles are consistent with previously reported data^6,9,10^.

As a negative control, the same test was applied to samples collected before vaccination (day 0 and day -7). In both cases, no genes were found to exceed the identified random expectation thresholds. In line with previous observations, also within the identified vaccine-induced gene modules the modulation was found to be more robust at day 1 compared to day 7. For day 1, the median number of subjects in which a gene was modulated was 30 (50% of the 60 subjects, IQR: 27-35), whereas at day 7 the median number was 25 (44% of the 57 subjects, IQR: 21-30).

### The magnitude of day 7 transcriptome response is associated with functional antibody response

Having identified the set of genes that are modulated by vaccination, we tested whether the extent of transcriptional modulation of these genes correlates with the humoral immune response. The percentage of modulated genes within each individual response profile was tested for correlation with subjects’ ability to produce functional antibodies using a receiver operating characteristic (ROC) curve. Subjects were stratified into *high* and *low responders* based on their day 70 influenza microneutralization titer response.

**Figure 5:**
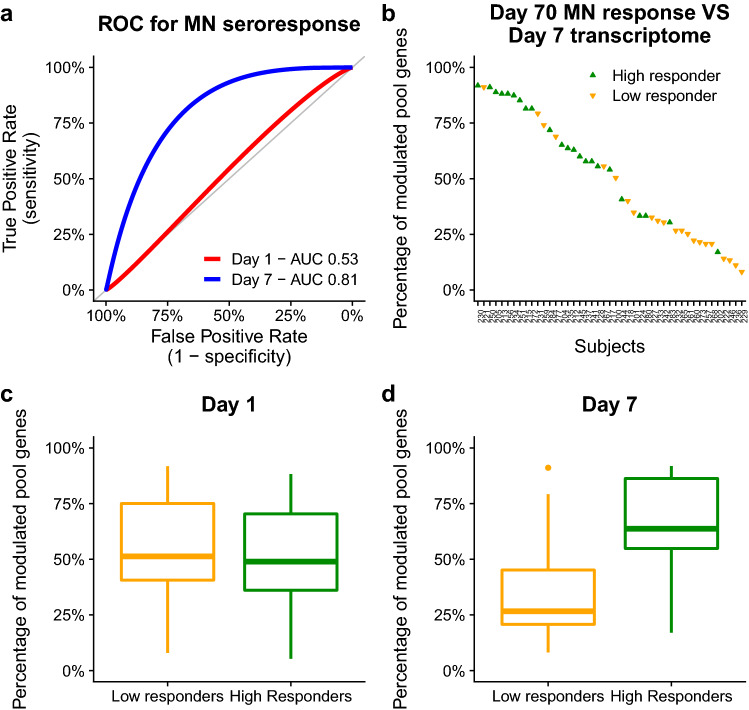
The day 7 vaccination-induced gene pool is associated with the magnitude of seroresponse detected through day 70 MicroNeutralization titers. (**a**) Smoothed ROC curves of the immunological response classification predictions inferred using the modulation percentages of the vaccination-induced gene pools calculated from the individual transcriptome response profiles. (**b**) Percentage of modulation in the day 7 vaccination-induced gene pool shown in relation to the immunological response classification based on day 70 Influenza MicroNeutralization titers. (**c,d**) Distributions of vaccination-induced gene pools modulation percentages grouped by immunological response classification.

Overall, the modulation of vaccination-induced gene pools was 51% (median, IQR: 36%-72%) for day 1 and 40% (median, IQR: 25%-68%) for day 7 (see Supplementary Figure S4). The ROC curve provided an area under the curve (AUC) of 0.53 and 0.81 respectively for day 1 and day 7 transcriptome response data (Fig. 5a), suggesting the existence of a correlation between the number of modulated genes at day 7 and the subsequent functional antibody production. Conversely, day 1 transcriptome data did not show any appreciable correlation. These evidences are in agreement with previous studies, which proposed that day 7 blood-derived signatures reflect the establishment of the humoral immune response^9,17,18^.

The correlation between the day 7 transcriptome response and subjects’ ability to produce neutralizing antibodies is shown in Figure 5b (see Supplementary Fig. S8 for day 1 data). The median number of modulated genes in *high responder* subjects was 63% (IQR: 55%-86%), while in *low responders* this number dropped to 27% (IQR: 20%-45%, Fig. 5c-d). This difference was statistically significant (*p*-value = 0.002, two-sided Kolmogorov-Smirnov tests followed by Benjamini-Hochberg correction for multiple testing). Further investigation identified 66 genes for which the modulation was significantly more frequent among the high responders than low responders (Fisher exact test, one sided, *p*-value $\le$ 0.05, Benjamini-Hochberg correction; see Supplementary Table S8). These included several genes participating in immune response functions. Overall, these data are indicative of the fact that subjects experiencing a higher transcriptional activity at day 7 are more likely to produce neutralizing antibodies.

To provide corroborative evidence in support to this finding we also tested whether the amount of vaccine specific IgG-secreting cells, measured using ELISpot assays targeted against the entire vaccine (seasonal or pandemic H1N1) 7 days post-vaccination, showed any correlation with the individual transcriptome response profiles. As observed with microneutralization titers, also the seasonal vaccine-specific B cell response showed association with day 7 transcriptome profiles (AUC = 0.74; supplementary Fig. S9). Conversely, the same trend was not observed with the pandemic H1N1-specific B cells (supplementary Fig.S10).

Finally, the same procedure was applied to evaluate the potential effect of subjects’ intrinsic characteristics (age, gender and pre-immune status) on their individual transcriptome response profile. None of the evaluated factors showed any appreciable effect (results relative to pre-existing vaccine-specific antibodies are shown in supplementary Fig. S11).

### Identification of genes associated with functional antibody response

**Figure 6:**
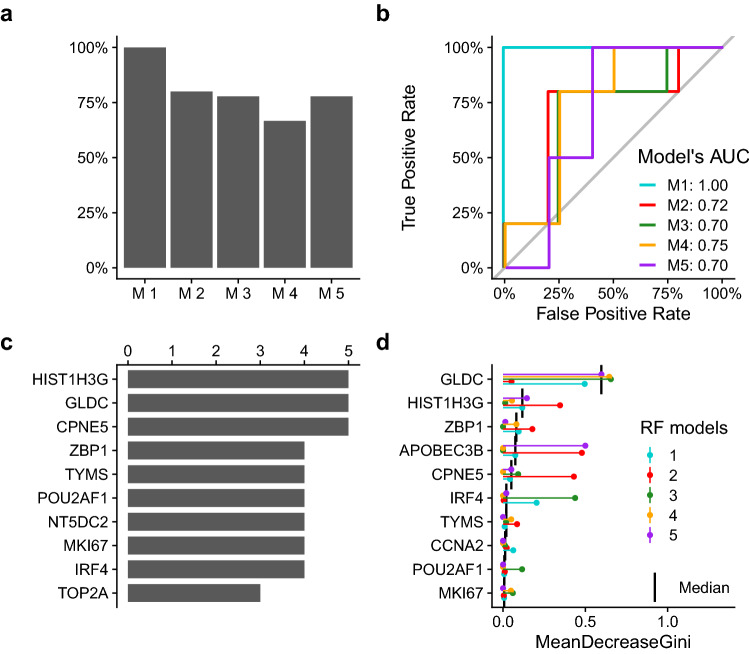
Guided Random Forest results. (**a**) Classification accuracy of the models (percentage of correctly classified subjects). (**b**) ROC curves and AUCs of the models. (**c**) Number of occurrences across the five models of the most informative genes (from each model, the top 10 most informative features are reported). The y-axis indicates the number of models in which a gene was observed in the top 10. (**d**) Top 10 most informative genes across GRF models ordered by median of the *mean decrease Gini* coefficients (higher values correspond to higher feature importance).

In order to get further insights into which genes were driving the observed correlation between the day 7 transcriptome response and day 70 microneutralization titers, we applied a 5-fold cross validated Guided Random Forest (GRF)^19^. Briefly, the strategy was to classify subjects into *high* or *low responders*, without prior knowledge of their microneutralization response, using the gene modulation data of the day 7 vaccination-induced gene pool as input. This approach was able to predict subjects’ functional antibody production with an 80% correct classification rate (min-max: 67%-100%, min-max AUCs: 70%-100%, Fig. 6a,b).

To understand which genes were most informative, the top 10 scoring genes from each of the five cross-validations were selected for further analysis. Three of these genes were selected by all five models, 6 genes were present in four out of five models and 1 was found in three models (Fig. 6c). Ranking of these genes by their median decrease Gini across all five models (Fig. 6d) highlighted *GLDC* as the most informative gene. This gene encodes a mitochondrial glycine decarboxylase, which was described to confer survival advantage to T-cells in hypoxic environments such as sites of infection^20,21^. Another top performing gene was *HIST1H3G*, which codes for a replication-dependent histone protein which is generally active during the S-phase^22^. We also observed *ZBP1*, a cytosolic DNA sensor which induces the DNA-mediated interferon response^23^ and *APOBEC3B*, a cytosine deaminase induced by viral infection^24^. Among these genes we also observed *IRF4* and *POU2AF1*, both of which are involved in maturation and differentiation of T and B cells^25–27^, respectively.

## Discussion

Human response to vaccination can be a heterogeneous phenomenon. While some of the factors contributing to this variation have been identified, others are still to be unraveled. Young and elderly individuals, for example, usually show a suboptimal response to vaccination due to their immune system being not fully developed or aged, respectively^28,29^. Several latent factors have also been proposed to impact vaccine responsiveness, the mechanisms through which they mediate their effect, however, are still subject of research^11^. It is generally recognized that this lack of knowledge on how immune responses to vaccination are generated is a critical barrier to the development of vaccines that are effective across diverse populations^30,31^.

To cope with the molecular heterogeneity characterizing a set of systemic diseases, Menche and coworkers^14^ devised a data analysis strategy for deriving personalized response profiles and overcome the inherent limitations of group-wise analysis methodologies. In this work, we presented an adaptation of this methodology, aimed at accommodating the analysis of datasets generated through repeated measures design studies and to provide an estimate of the robustness of the observed gene perturbations.

The individual-subject response profiles derived from healthy adults following influenza vaccination showed a high degree of heterogeneity, both in terms of total number of modulated genes within each individual and in the consistency of modulation of a same gene across multiple subjects. Day 1 individual response profiles were generally more consistent than those observed 7 days post-vaccination. This may be explained by the fact that differences among subjects add up during the chain of events linking the innate and adaptive arms of the immune response. To our surprise, however, no gene was found to be consistently modulated in all study subjects, regardless of the sampling time point.

In principle, the high heterogeneity observed among different individuals could have arisen from technical variation, random noise or a combination of the two. In such case, functional enrichment analysis of the individual response profiles would have failed in identifying molecular pathways that are consistently enriched across multiple individuals. Our results, instead, showed consistent enrichment of a number of molecular pathways, covering immune related functions, that were previously described to be involved in the immune response to influenza vaccination. These include cytokine signaling and Interferon-mediated antiviral response for day 1 response profiles and transcripts involved in B lymphocyte proliferation and differentiation into antibody-producing cells for day 7 profiles^6,9^. These evidences point to the conclusion that influenza vaccination consistently activates these biological processes, while the observed heterogeneity in the individual transcriptome response profiles represents the molecular diversity of such functional perturbations.

One of the main areas of interest for modern vaccinology studies is that of identifying early cellular or molecular biomarkers that are able to predict the quality and magnitude of the adaptive response to vaccination. After deriving subjects’ individual response profiles, we found that the number of modulated genes within each individual subject 7 days post-vaccination was correlated with the magnitude of vaccine-specific functional antibody response. Given the functional analysis of day 7 transcriptome responses, which highlighted an enrichment of genes involved in B-cell and plasmablasts response, it could be assumed that the ability of the influenza vaccine to elicit a plasmablasts response at day 7 is linked with the downstream production of functional antibodies. We also investigated on which genes are most strongly associated with a high antibody response through a machine learning approach. This analysis identified genes involved in protein production (*GLDC* and *HIST1H3G*) and antiviral response (*APOBEC3B* and *ZBP1*). These evidences, which recall classical notions of immunology, represent a corroborative evidence for the relevance of our methodology. Importantly, results on the identified genes, which could potentially serve as predictive biomarkers, should be interpreted with caution until further validation through independent analyses or, ideally, experimental testing.

Overall, the present study allowed for a deeper characterization of the transcriptional response induced by influenza vaccination in humans. We argue that the improved characterization of the intersubject heterogeneity offered by this approach will help driving the improvement and optimization of both new and established vaccines. The ability to identify genes that are predictive of functional antibody production following vaccination, along with the fraction of subjects in which they are modulated, can allow for better predictions on the real-world effectiveness of vaccines. Furthermore, individual subject analysis approaches could help the improvement of vaccines targeting specific subpopulations, like the elderly or immunocompromised individuals.

Finally, we envision that the application of this methodology to multiple vaccine studies and datasets will be foundational to expand our understanding of vaccines’ mode of action. Our methodology was designed to be readily generalizable to all repeated measures experiments and adaptable to different types of data.

## Methods

### Study dataset

The present study was based on data previously reported by Tsang *et al.* 2014. In that study (Clinicaltrials.gov NCT01191853), 63 healthy adults were vaccinated with a single dose of both the 2009 FLUVIRIN seasonal influenza (Novartis) and H1N1 pandemic (Sanofi-Aventis) vaccines, both without adjuvant. Pre-processed Affymetrix microarray data were downloaded from the GEO database using the GSE47353 accession number. Control and non-protein coding probe sets were filtered out and probe sets mapping onto the same gene were collapsed by computing their geometric average fluorescence intensity. To limit the dataset dimensionality, the median absolute deviation across the dataset was computed for each transcript and the 5000 most variable ones were retained for further analysis. Antibody response data were collected from the original manuscript^18^.

### Group-wise transcriptome data analysis

Group-wise comparisons of transcript abundance values were performed using a two-sided, paired Wilcoxon signed-rank test. *p*-values were corrected using the Benjamini-Hochberg procedure. Genes showing an absolute log$_{2}$ fold-change from baseline $\ge 0.5$ and an adjusted *p*-value $\le 0.05$ were considered significantly modulated.

### Analytical framework for deriving individual transcriptome response profiles

Transcriptome response data were cleaned from any subject-specific effect through a mixed-effect model. Transcript abundance values (*v*) were set as the dependent variable, the sampling time points (*t*) as the fixed effect and the subject identity (*s*) as the random effect [lmer$(v \sim t + (1|s))$]. Fitted random effect components were then subtracted from the original data.

Gene modulation in individual subjects was assessed by comparing the transcript abundance of each single gene at a specific time point to the respective abundance values observed in the study population before vaccination (reference sample). To limit the effect of outlying values and to assess the robustness of the response, this comparison was repeated 1000 times over bootstrapped reference distributions obtained by randomly selecting 2/3 of the study subjects. If a test gene abundance differed from that of the reference distribution over a predefined threshold (median ± 2.5 median absolute deviations), the gene was assumed to test positive. Genes testing positively over $\ge 75\%$ of bootstrap iterations were regarded as being modulated by vaccination. Genes were tested for both up- and down-regulation from baseline. This approach produced individual transcriptome response profiles consisting of arrays of categorical gene modulation values ($0 =$ no modulation, $1 =$ up-regulation, $- 1 =$ down-regulation). One response profile was generated for each subject and time point combination.

### Gene set Enrichment Analysis

A curated list of canonical biological pathways (C2 collection, v6.1) was obtained from the Molecular Signatures Database https://www.gsea-msigdb.org/gsea/msigdb/index.jsp;^32–34^). Pathways with less than 10, or more than 200 mapped genes were excluded from the analysis, providing a total of 437 gene sets and 2369 mapped genes. Functional enrichment was computed using the Fisher’s exact test followed by the Benjamini-Hochberg *p*-value correction for multiple testing. For each individual response profile, the proportion of modulated genes within a given pathway was compared with the proportion of modulation of genes not included in that pathway. Pathways scoring an adjusted *p*-value $\le 0.05$ were considered significantly enriched. The overall vaccination effect was then assessed by counting, for each pathway, in how many subjects it was found to be enriched.

To test whether the observed pathway enrichments were indicative of a true biological signal, as opposed to random observation, we tested whether genes within each given pathway were more consistently modulated than an equally sized set of randomly selected genes. The Jaccard similarity index^35^ was computed for all pairwise subject combinations to assess the proportion of genes being modulated across multiple subjects. This metric was computed twice, once using the genes belonging to a given pathway, and another time using an equally sized set of randomly selected genes (the test on the randomly selected genes was bootstrapped 1000 times). If genes’ modulation within a test pathway was more pervasive ($\ge 95\%$ quantile of the control values) than that of randomly selected genes, that signal was assumed to be biologically meaningful.

### Definition of vaccination-induced gene pools

Starting from the individual response profiles, the pool of genes modulated by vaccination was determined based on the comparison with the random probability of a gene to be modulated across multiple subjects. This null probability was computed using a binomial distribution, according to a previously established approach^14^. Briefly, a null model is established where each subject has *g* modulated genes drawn randomly from all *G* genes. The probability for one gene to be modulated in *k* out of *n* subjects can then be calculated via the binomial distribution, where $p = \frac{g}{G}$.

1 $$\begin{aligned} f(k;n,p) = \text {Pr}(X=k) = \begin{pmatrix} n \\ k \end{pmatrix} p^k (1 - p)^{n-k} \end{aligned}$$

Setting *g* as the mean number of genes observed across the individual profiles at a given time point, it is possible to derive the histogram of the number of subjects in which a given gene is found to be co-modulated by random chance by multiplying $G \times (f;n,p)$.

Based on this approach, we computed 1000 random binomial distributions using $G = 5000$, $n =$ number of available subjects for each specific time point and $p = \frac{g}{G}$. For each iteration, the minimum number of subjects ($X_{\min }$) in which the resulting number of commonly modulated genes was zero was retained, providing a pool of 1000 $X_{\min }$ values. We then set the threshold number of subjects (*X*) for inclusion in the vaccination-induced gene pool as the 95th percentile of the $X_{\min }$ values distribution. This allowed to identify genes with a probability $\lesssim$ 5% of being co-modulated across $\ge X$ subjects. The resulting *X* values were 25 and 19 subjects for day 1 and day 7 responses, respectively. Therefore, genes found to be co-modulated in $\ge$ 25 subjects for day 1 and $\ge$ 19 subjects for day 7 were regarded as being specifically modulated by vaccination.

### Identification of transcriptional signatures associated with antibody response

Subjects were classified into *high* and *low responders* based on their functional antibody response. The day 70 fold-increase in influenza MicroNeutralization titer, the maximum value across the different strains represented in the vaccine, was used as response measure. Subjects for which the response was above or below the median of the distribution were respectively tagged as *high* and *low responders*. The percentage of modulated genes within each individual response profile was then used as discriminant variable for *high* and *low responder* subjects using a receiver operating characteristic curve. The area under the curve was used as measure of the classification performance.

In order to identify those genes that were contributing the most to the *high*/*low responder* subjects’ classification, we applied a guided random forest classifier (GRF)^19^ combined with a five-fold cross-validation. This task was performed using the *RRF* R package with the following parameters: *ntree* = 30000 and *mtry* = 4. The penalty score of the GRF^19^ was set to maximum ($\gamma = 1$). Variables (genes) importance was imputed based on the mean decrease Gini score obtained across the five cross-validated GRF models.

## Supplementary information

Supplementary Information 1.

Supplementary Information 2.

Supplementary Information 3.

Supplementary Information 4.

Supplementary Information 5.

## References

[1] Krammer, F. *et al.**Influenza. Nat. Rev.* **4**, 1. 10.1016/B978-0-323-44887-1.00027-4 (2018).

[2] Sellers SA, Hagan RS, Hayden FG, Fischer WA (2017). The hidden burden of influenza: A review of the extra-pulmonary complications of influenza infection. Influen. Respirat. Viruses.

[3] Thompson WW (2003). Mortality associated with influenza and respiratory syncytial virus in the United States. JAMA.

[4] Fiore, A. E. *et al.* Prevention and control of influenza with vaccines: recommendations of the Advisory Committee on Immunization Practices (ACIP). *MMWR Recomm Rep.* **59**, 1 (2010). 20689501

[5] Paules CI, Fauci AS (2019). Influenza Vaccines: Good, but We Can Do Better. J. Infect. Dis..

[6] Nakaya HI (2011). Systems biology of vaccination for seasonal influenza in humans. Nat. Immunol..

[7] Nakaya HI (2015). Systems analysis of immunity to influenza vaccination across multiple years and in diverse populations reveals shared molecular signatures. Immunity.

[8] Parra-Rojas, C., von Messling, V. & Hernandez-Vargas, E. A. Adjuvanted influenza vaccine dynamics. *Sci. Rep.* **9**, 1–15. 10.1038/s41598-018-36426-9 (2019). 10.1038/s41598-018-36426-9 PMC6329778 30635599

[9] Bucasas KL (2011). Early patterns of gene expression correlate with the humoral immune response to influenza vaccination in humans. J. Infect. Dis..

[10] Obermoser G (2013). Systems scale interactive exploration reveals quantitative and qualitative differences in response to influenza and pneumococcal vaccines. Immunity.

[11] Rappuoli, R., Siena, E. & Finco, O. Will systems biology deliver its promise and contribute to the development of new or improved vaccines?: Systems biology views of vaccine innate and adaptive immunity. *Cold Spring Harbor Perspect. Biol.* **10**, 10.1101/cshperspect.a029256 (2017). 10.1101/cshperspect.a029256 PMC6071491 29038117

[12] Brodin P, Davis MM (2017). Human immune system variation. Nat. Rev. Immunol..

[13] Ahmed E, Hashish A (2006). On modelling the immune system as a complex system. Theory Biosci..

[14] Menche, J. *et al.* Integrating personalized gene expression profiles into predictive disease-associated gene pools. *npj Systems Biology and Applications* **3**, 1. 10.1038/s41540-017-0009-0 (2017). 10.1038/s41540-017-0009-0 PMC5445628 28649437

[15] Dufour JH (2002). IFN-gamma-Inducible Protein 10 (IP-10; CXCL10)-Deficient Mice Reveal a Role for IP-10 in Effector T Cell Generation and Trafficking. J. Immunol..

[16] Sobolev O (2016). Adjuvanted influenza-H1N1 vaccination reveals lymphoid signatures of age-dependent early responses and of clinical adverse events. Nat. Immunol..

[17] Bonilla FA, Oettgen HC (2010). Adaptive immunity. J. Allergy Clin. Immunol..

[18] Tsang JS (2014). Global analyses of human immune variation reveal baseline predictors of postvaccination responses. Cell.

[19] Deng H (2013). Guided Random Forest in the RRF Package. CoRR.

[20] Buck MD, O’Sullivan D, Pearce EL (2015). T cell metabolism drives immunity. J. Experim. Med..

[21] Pearce EL, Pearce EJ (2013). Metabolic pathways in immune cell activation and quiescence. Immunity.

[22] Marzluff WF, Gongidi P, Woods KR, Jin J, Maltais LJ (2002). The human and mouse replication-dependent histone genes. Genomics.

[23] Takaoka A (2007). DAI (DLM-1/ZBP1) is a cytosolic DNA sensor and an activator of innate immune response. Nature.

[24] McCann JL (2019). The DNA deaminase APOBEC3B interacts with the cell-cycle protein CDK4 and disrupts CDK4-mediated nuclear import of Cyclin D1. J. Biol. Chem..

[25] Yao, S. *et al.* Interferon regulatory factor 4 sustains CD8+T cell expansion and effector differentiation. *Immunity* **39**, 833–845, 10.1016/j.immuni.2013.10.007 (2013). 10.1016/j.immuni.2013.10.007 PMC3855863 24211184

[26] Krishnamoorthy V (2017). The IRF4 gene regulatory module functions as a read–write integrator to dynamically coordinate T helper cell fate. Immunity.

[27] Massa S, Junker S, Schubart K, Matthias G, Matthias P (2003). The OBF-1 gene locus confers B cell-specific transcription by restricting the ubiquitous activity of its promoter. Eur. J. Immunol..

[28] Mugitani A (2014). Immunogenicity of the trivalent inactivated influenza vaccine in young children less than 4 years of age, with a focus on age and baseline antibodies. Clin. Vaccine Immunol..

[29] Lord JM (2013). The effect of aging of the immune system on vaccination responses. Hum. Vaccines Immunother..

[30] Davis, M. M. & Tato, C. M. Will systems biology deliver its promise and contribute to the development of new or improved vaccines?. *Cold Spring. Harbor Perspect. Biol.* **10**. 10.1101/cshperspect.a028886 (2018). 10.1101/cshperspect.a028886 PMC5902661 29038119

[31] Pezeshki A, Ovsyannikova IG, McKinney BA, Poland GA, Kennedy RB (2019). The role of systems biology approaches in determining molecular signatures for the development of more effective vaccines. Expert Rev. Vaccines.

[32] Subramanian, A. *et al.* Gene set enrichment analysis: A knowledge-based approach for interpreting genome-wide expression profiles. *Proc. Natl. Acad. Sci.* **102**, 15545–15550, 10.1073/pnas.0506580102 (2005). 10.1073/pnas.0506580102 PMC1239896 16199517

[33] Liberzon, A. *et al.* Molecular signatures database (MSigDB) 3.0. *Bioinformatics* **27**, 1739–1740, 10.1093/bioinformatics/btr260 (2011). 10.1093/bioinformatics/btr260 PMC3106198 21546393

[34] Liberzon A (2015). The molecular signatures database hallmark gene set collection. Cell Syst..

[35] Jaccard P (1912). The distribution of the flora in the alpine zone. New Phytol..

