# Supplementary material for: Systematic characterization of human response to H1N1 influenza vaccination through the construction and integration of personalized transcriptome response profiles
Source: Sci Rep. 2021 Oct 21;11:20821. doi: 10.1038/s41598-021-99870-0 (PMC8531369; doi:10.1038/s41598-021-99870-0)
Supplement: Supplementary file 4 — Supplementary Information 4. [file 41598_2021_99870_MOESM4_ESM.pdf]

Systematic characterization of human response to H1N1 influenza vaccination through the construction and integration of personalized transcriptome response profiles

Carlo De Intinis[1,2], Margherita Bodini[2], Denise Maffione[2,†], Laurane De Mot[3,‡], Margherita Coccia[3], Duccio Medini[2,§], and Emilio Siena[2,*]

[1]University of Turin, 10124 Turin, Italy
[2]GSK, 53100 Siena, Italy
[3]GSK, 1330 Rixensart, Belgium
[†]Currently at AizoOn, 10146 Turin, Italy
[‡]Currently at Clarivate Analytics, 2600 Berchem, Belgium
[§]Currently at Toscana Life Sciences, 53100 Siena, Italy
[*]Corresponding author Email: emilio.x.siena@gsk.com

Supplementary table S1: Day 1 differentially expressed genes from group-wise analysis

Gene	MFC	-log10 adjusted p-value (BH)	Day 0 mean	Day 1 mean	Day 0 SD	Day 1 SD	Number of subjects with Day 1 gene expression value within 1 SD of Day 0	Number of subjects with Day 0 gene expression value higher than Day 1 mean
ANKRD22	1.437	6.284	5.855	7.247	0.935	1.343	25	2
HBEGF	1.258	6.775	7.97	9.257	0.731	0.855	9	5
G0S2	1.077	6.985	8.105	9.212	0.669	0.682	11	2
IL1B	1.047	5.768	7.787	8.872	0.837	0.813	18	6
CXCL10	0.962	6.124	6.378	7.294	0.896	1.169	30	5
PTGS2	0.884	6.183	8.559	9.46	0.821	0.781	19	7
SERPING1	0.862	6.585	6.813	7.654	0.462	0.849	22	2
SOCS3	0.789	6.985	7.591	8.399	0.46	0.517	10	3
NR4A3	0.74	6.337	6.819	7.578	0.482	0.584	13	5
B3GNT5	0.728	6.124	6.951	7.699	0.678	0.749	23	9
OSM	0.723	6.562	7.029	7.756	0.363	0.531	8	0
IDO1	0.709	5.733	5.837	6.516	0.508	0.89	28	2
IFIT2	0.704	6.523	6.5	7.179	0.572	0.69	29	6
NR4A2	0.695	6.072	8.929	9.659	0.646	0.554	19	8
AREG	0.657	3.027	8.362	9.018	0.916	0.86	34	15
RGS1	0.657	5.341	7.374	8.027	0.607	0.657	27	8
PLK2	0.657	5.396	5.876	6.552	0.475	0.639	16	4
CD83	0.654	6.341	8.362	9.026	0.439	0.493	11	5
FOSB	0.619	5.477	7.846	8.502	0.641	0.642	28	6
JUN	0.619	6.985	8.375	8.999	0.478	0.365	24	5
CA1	0.607	5.706	7.132	7.718	1.246	1.337	42	14
GBP4	0.604	6.284	8.719	9.309	0.384	0.519	18	2
KLF4	0.596	6.985	8.178	8.789	0.385	0.393	15	3
FFAR2	0.596	6.29	5.754	6.354	0.457	0.652	27	3
FOS	0.589	6.985	11.389	11.988	0.432	0.343	15	4
CXCL8	0.571	3.917	9.497	10.09	0.822	0.708	32	13
PFKFB3	0.562	5.765	8.836	9.42	0.402	0.51	18	8
STAT1	0.558	6.747	10.441	10.996	0.337	0.427	18	2
LAP3	0.555	6.57	8.249	8.807	0.354	0.475	23	3
NAMPT	0.552	6.178	10.78	11.343	0.506	0.5	22	8
ATF3	0.552	6.985	5.994	6.551	0.237	0.379	10	0
CLEC6A	0.541	5.706	7.567	8.071	0.613	0.607	27	11
TRIB1	0.539	6.127	8.319	8.878	0.482	0.5	20	7
EPSTI1	0.534	6.415	7.743	8.264	0.479	0.541	28	7
RIPK2	0.52	6.777	8.761	9.281	0.276	0.399	10	2
CCL2	0.512	5.429	6.263	6.759	0.479	0.898	36	9
EREG	0.507	5.234	7.39	7.922	0.518	0.608	27	8
CCDC59	0.506	6.471	8.742	9.232	0.421	0.488	27	6
HCAR3	0.504	4.794	5.63	6.142	0.516	0.592	25	9
PMAIP1	0.503	6.172	7.947	8.456	0.33	0.422	12	2
NFIL3	0.502	5.879	8.556	9.061	0.42	0.434	19	7
EGR2	0.501	3.555	8.299	8.818	0.817	0.854	35	11
DDIT3	0.501	6.65	7.681	8.193	0.253	0.36	13	2

Supplementary table S2: Day 7 differentially expressed genes from group-wise analysis

Gene	MFC	-log10 adjusted p-value (BH)	Day 0 mean	Day 7 mean	Day 0 SD	Day 7 SD	Number of subjects with Day 1 gene expression value within 1 SD of Day 0	Number of subjects with Day 0 gene expression value higher than Day 1 mean
TNFRSF17	1.387	5.078	5.364	6.677	0.641	1.041	17	1
GPRC5D	1.054	4.91	6.471	7.476	0.644	1.011	20	3
JCHAIN	0.979	5.078	10.374	11.349	0.575	0.689	16	1
MZB1	0.733	5.078	6.911	7.623	0.381	0.557	18	1
CD38	0.676	4.958	8.626	9.264	0.45	0.56	22	2
APOBEC3B	0.674	5.078	7.67	8.318	0.441	0.609	29	3
ITM2C	0.649	4.959	8.891	9.512	0.359	0.513	22	1
CAV1	0.616	4.935	5.066	5.651	0.377	0.557	26	1
DENND5B	0.547	4.388	6.192	6.717	0.444	0.579	29	3
KLHL14	0.546	3.972	6.346	6.876	0.499	0.642	27	4
ELL2	0.534	4.904	7.018	7.532	0.318	0.543	23	1
NT5DC2	0.531	4.796	7.111	7.619	0.354	0.583	26	2
MYBL2	0.528	4.959	6.743	7.241	0.393	0.532	29	2
HIST1H3B	0.51	3.961	7.108	7.598	0.504	0.593	30	6

Supplementary table S3: Number of subjects with enriched pathway at Day -7

Pathway Name	Subjects
KEGG taste transduction	5
REACTOME cell cycle	5
REACTOME rna pol i transcription	5
REACTOME chromosome maintenance	5
REACTOME rna pol i promoter opening	5
REACTOME meiotic recombination	5
REACTOME meiotic synapsis	5
REACTOME amyloids	5
KEGG systemic lupus erythematosus	4
PID ap1 pathway	4
REACTOME meiosis	4
REACTOME generic transcription pathway	4
REACTOME rna pol i rna pol iii and mitochondrial transcription	4
REACTOME deposition of new cenpa containing nucleosomes at the centromere	4
REACTOME packaging of telomere ends	4
REACTOME telomere maintenance	4
PID nfat tfpathway	3
PID atf2 pathway	3
PID hif1 tfpathway	3
REACTOME cell cycle mitotic	3

Supplementary table S4: Number of subjects with enriched pathway at Day 0

Pathway Name	Subjects
REACTOME olfactory signaling pathway	4
REACTOME amyloids	4
REACTOME packaging of telomere ends	4
KEGG focal adhesion	3
REACTOME deposition of new cenpa containing nucleosomes at the centromere	3
REACTOME rna pol i promoter opening	3
REACTOME interferon alpha beta signaling	3
KEGG ribosome	2
KEGG olfactory transduction	2
KEGG asthma	2
KEGG systemic lupus erythematosus	2
REACTOME meiosis	2
REACTOME immunoregulatory interactions between a lymphoid and a non lymphoid cell	2
REACTOME srp dependent cotranslational protein targeting to membrane	2
REACTOME mhc class ii antigen presentation	2
REACTOME tcr signaling	2
REACTOME generation of second messenger molecules	2
REACTOME generic transcription pathway	2
REACTOME rna pol i transcription	2
REACTOME transcription	2

Supplementary table S5: Number of subjects with enriched pathway at Day 1

Pathway Name	Subjects
REACTOME cytokine signaling in immune system	28
REACTOME interferon gamma signaling	27
REACTOME interferon signaling	26
REACTOME interferon alpha beta signaling	20
PID il12 2pathway	19
REACTOME adaptive immune system	11
PID atf2 pathway	9
PID ap1 pathway	9
PID il6 7 pathway	9
KEGG graft versus host disease	8
REACTOME tcr signaling	8
PID il23 pathway	7
REACTOME activation of nf kappab in b cells	7
REACTOME signaling by the b cell receptor bcr	7
REACTOME regulation of ornithine decarboxylase odc	7
REACTOME p53 dependent g1 dna damage response	7
REACTOME apc c cdh1 mediated degradation of cdc20 and other apc c cdh1 targeted proteins in late mitosis early g1	7
REACTOME scf beta trcp mediated degradation of emi1	7
KEGG proteasome	6
PID reg gr pathway	6

Supplementary table S6: Number of subjects with enriched pathway at Day 7

Pathway Name	Subjects
REACTOME cell cycle	17
REACTOME rna pol i promoter opening	17
REACTOME amyloids	17
KEGG systemic lupus erythematosus	16
REACTOME meiotic recombination	16
REACTOME rna pol i transcription	15
REACTOME packaging of telomere ends	15
REACTOME meiosis	14
REACTOME rna pol i rna pol iii and mitochondrial transcription	14
REACTOME deposition of new cenpa containing nucleosomes at the centromere	14
REACTOME telomere maintenance	14
PID e2f pathway	13
REACTOME cell cycle mitotic	12
REACTOME transcription	12
REACTOME g0 and early g1	11
REACTOME meiotic synapsis	11
REACTOME chromosome maintenance	9
REACTOME asparagine n linked glycosylation	8
REACTOME mitotic g1 g1 s phases	6
REACTOME dna replication	6

Supplementary table S7: Number of subjects with enriched pathway at Day 70

Pathway Name	Subjects
KEGG taste transduction	7
KEGG systemic lupus erythematosus	5
REACTOME cell cycle	5
REACTOME transcription	5
REACTOME rna pol i promoter opening	5
REACTOME amyloids	5
PID e2f pathway	4
REACTOME meiosis	4
REACTOME rna pol i transcription	4
REACTOME cell cycle mitotic	4
REACTOME g1 s transition	4
REACTOME mitotic g1 g1 s phases	4
REACTOME rna pol i rna pol iii and mitochondrial transcription	4
REACTOME chromosome maintenance	4
REACTOME deposition of new cenpa containing nucleosomes at the centromere	4
REACTOME meiotic recombination	4
REACTOME meiotic synapsis	4
REACTOME packaging of telomere ends	4
REACTOME telomere maintenance	4
KEGG cell cycle	3

Supplementary table S8: Subpool of genes modulated specifically in the high reponder class

Gene	p-value
APOBEC3B	0.001
IRF4	0.001
HIST1H3G	0.001
GLDC	0.001
MKI67	0.002
POU2AF1	0.002
TOP2A	0.002
TYMS	0.002
NT5DC2	0.002
CD38	0.002
CCNA2	0.002
CPNE5	0.002
ITM2C	0.003
CHAC2	0.004
SEC11C	0.004
ZBP1	0.005
HIST2H3D	0.006
ELL2	0.006
KIF11	0.006
DLGAP5	0.006
SHCBP1	0.006
MYO1D	0.006
RRM2	0.006
SEL1L3	0.006
HIST1H2BM	0.006
HIST1H3B	0.006
HIST1H3F	0.006
HIST1H3J	0.006
ESCO2	0.006
GPRC5D	0.009
CAV1	0.009
BUB1	0.011
TPX2	0.011

Gene	p-value
UGT2B17	0.011
HIST1H2AM	0.011
MAN1A1	0.014
JCHAIN	0.017
CDK1	0.018
LMAN1	0.018
SLAMF7	0.022
LAMC1	0.022
DTL	0.022
HRASLS2	0.022
DENND5B	0.022
CCNB2	0.022
KLHL14	0.022
ERLEC1	0.022
PDIA6	0.022
DERL3	0.022
MANEA	0.022
HIST1H1B	0.022
MOXD1	0.022
PNOC	0.022
GGH	0.022
MYBL2	0.027
PHGDH	0.037
LAX1	0.037
ASPM	0.037
KNL1	0.037
LARP1B	0.037
FAM46C	0.039
SLC35F2	0.039
CDC6	0.039
COBLL1	0.039
FBXO16	0.039
BHLHE41	0.048
