# Supplementary material for: Systematic characterization of human response to H1N1 influenza vaccination through the construction and integration of personalized transcriptome response profiles
Source: Sci Rep. 2021 Oct 21;11:20821. doi: 10.1038/s41598-021-99870-0 (PMC8531369; doi:10.1038/s41598-021-99870-0)
Supplement: Supplementary file 5 — Supplementary Information 5. [file 41598_2021_99870_MOESM5_ESM.pdf]

Systematic characterization of human response to H1N1 influenza vaccination through the construction and integration of personalized transcriptome response profiles

Carlo De Intinis[1,2], Margherita Bodini[2], Denise Maffione[2,†], Laurane De Mot[3,‡], Margherita Coccia[3], Duccio Medini[2,§], and Emilio Siena[2,*]

[1]University of Turin, 10124 Turin, Italy
[2]GSK, 53100 Siena, Italy
[3]GSK, 1330 Rixensart, Belgium
[†]Currently at AizoOn, 10146 Turin, Italy
[‡]Currently at Clarivate Analytics, 2600 Berchem, Belgium
[§]Currently at Toscana Life Sciences, 53100 Siena, Italy
[*]Corresponding author Email: emilio.x.siena@gsk.com

Supplementary figure S1: Differentially expressed genes found via group-wise analysis

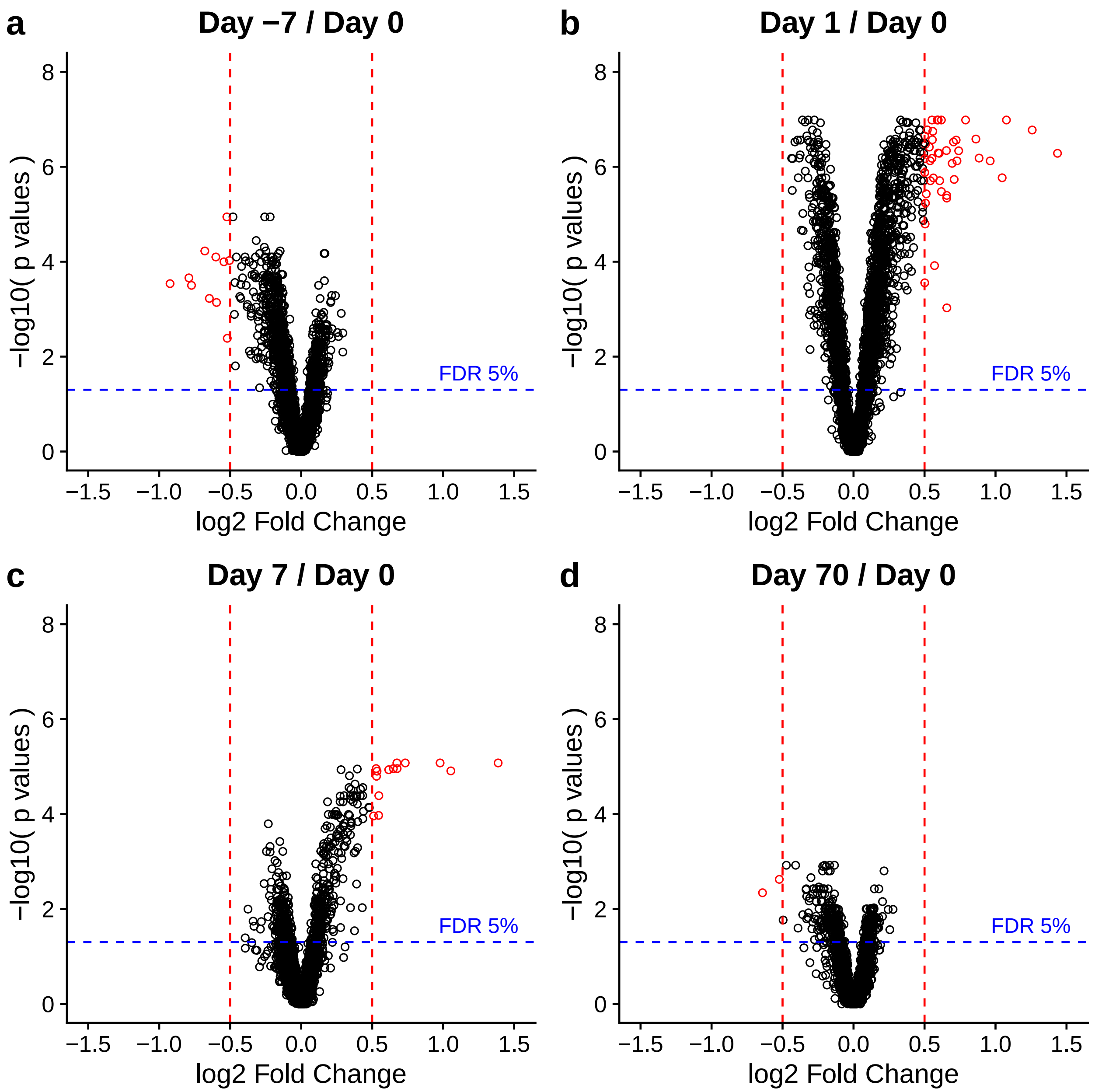

Figure S1. Group-wise DEGs analysis executed on various time points / day 0 gene expression values (detected DEGs are shown in red). The dashed lines show the cutoffs for calling a gene differentially expressed: log2 Fold Change ± 0.5 (red lines), p-value < 0.05 (two-tailed, paired Wilcoxon signed-rank test, blue line).

Supplementary figure S2: Percentage of subjects in which differentially expressed genes from group-wise analysis show expression values within 1SD of the control time point

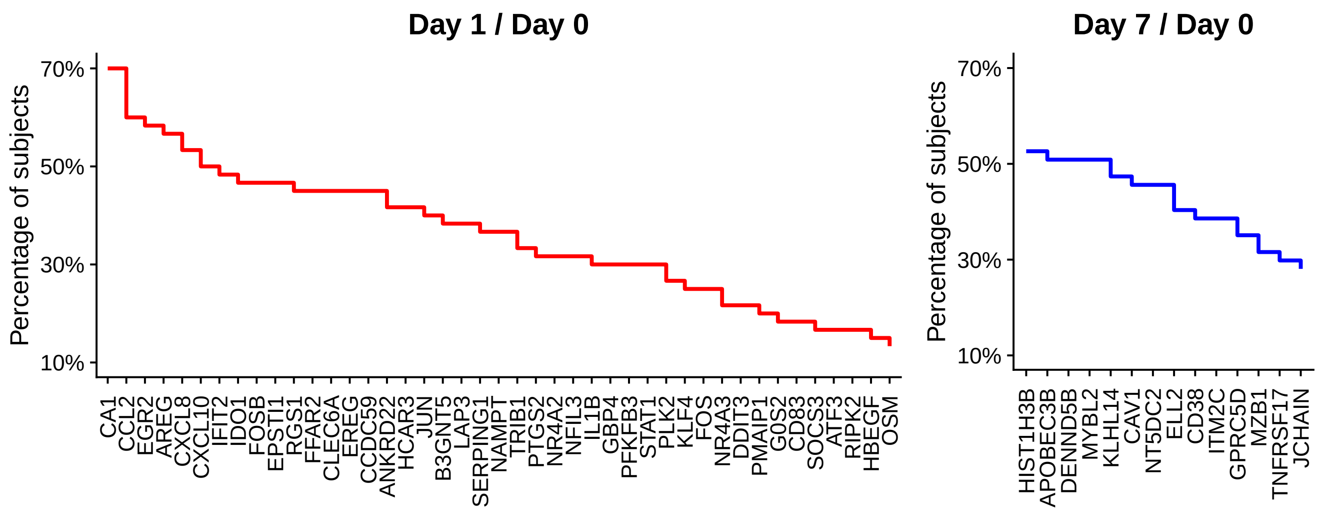

Figure S2. Reverse cumulative distributions of percentages of subjects in which differentially expressed genes from group-wise analysis show expression values within 1 standard deviation of the control time point.

Supplementary figure S3: Differentially expressed genes from group-wise analysis found in individual subject transcriptome profiles

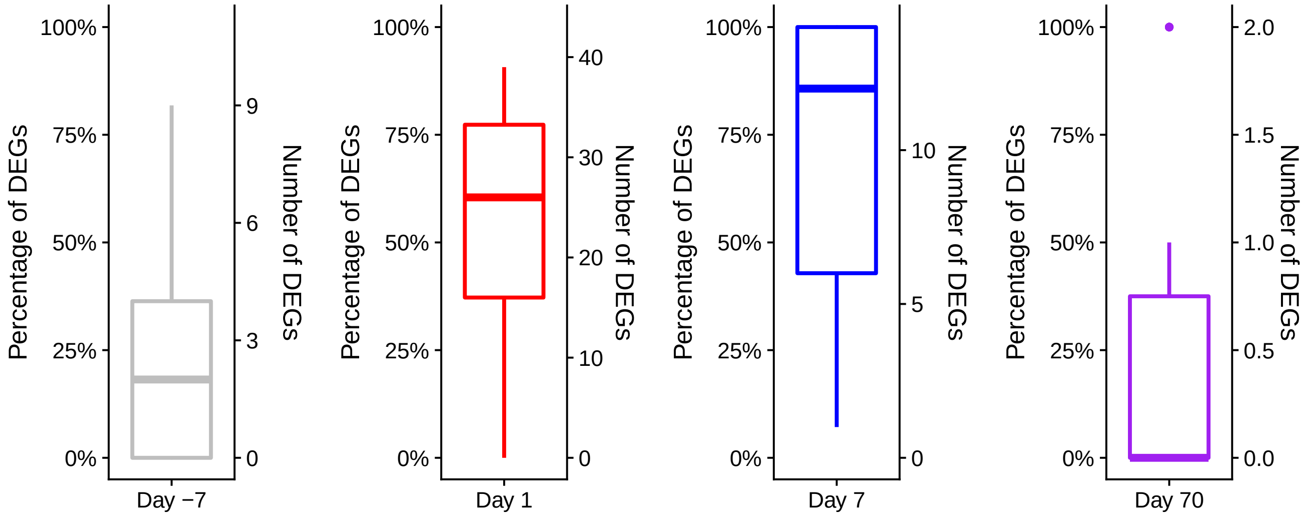

Figure S3. Differentially expressed genes from group-wise analysis that are also found within individual subject transcriptome profiles. *Right y-axes*: number of DEGs from group-wise analyses found in each individual transcriptome response profile. *Left y-axes*: percentage of DEGs from group-wise analysis found in each individual transcriptome response profile.

Supplementary figure S4: Modulation of vaccination-induced gene pools

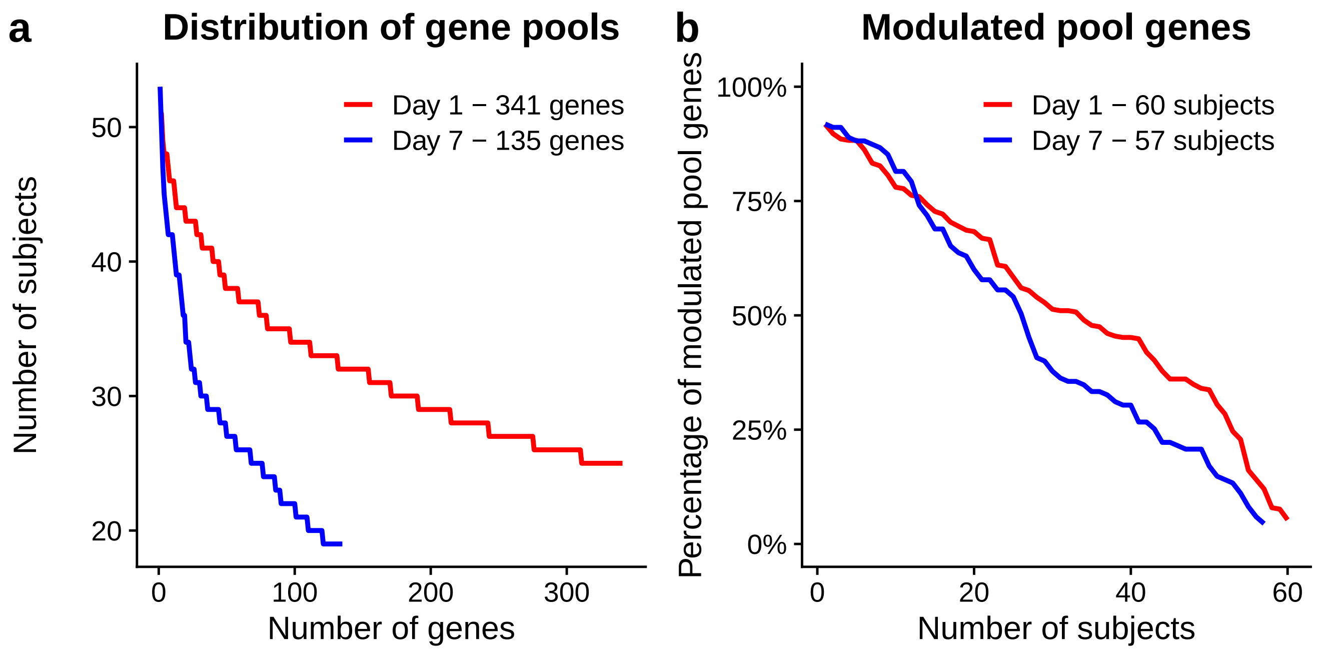

Figure S4. **a** Reverse cumulative distributions of the number of subjects showing modulation of the genes belonging to the day 1 and day 7 pools. Y-axis: subjects in which *n* genes (on the X-axis) are observed. Subjects are ranked in a decreasing Y-axis value manner, disregarding subject identity across time points. **b** Percentage of modulation observed in the pools across each subject. Subjects are ranked in a decreasing Y-axis value manner, disregarding subject identity across time points.

Supplementary figure S5: Most frequently enriched pathways within day 1 individual subject transcriptomes (all subjects, top 10: 2 to 10)

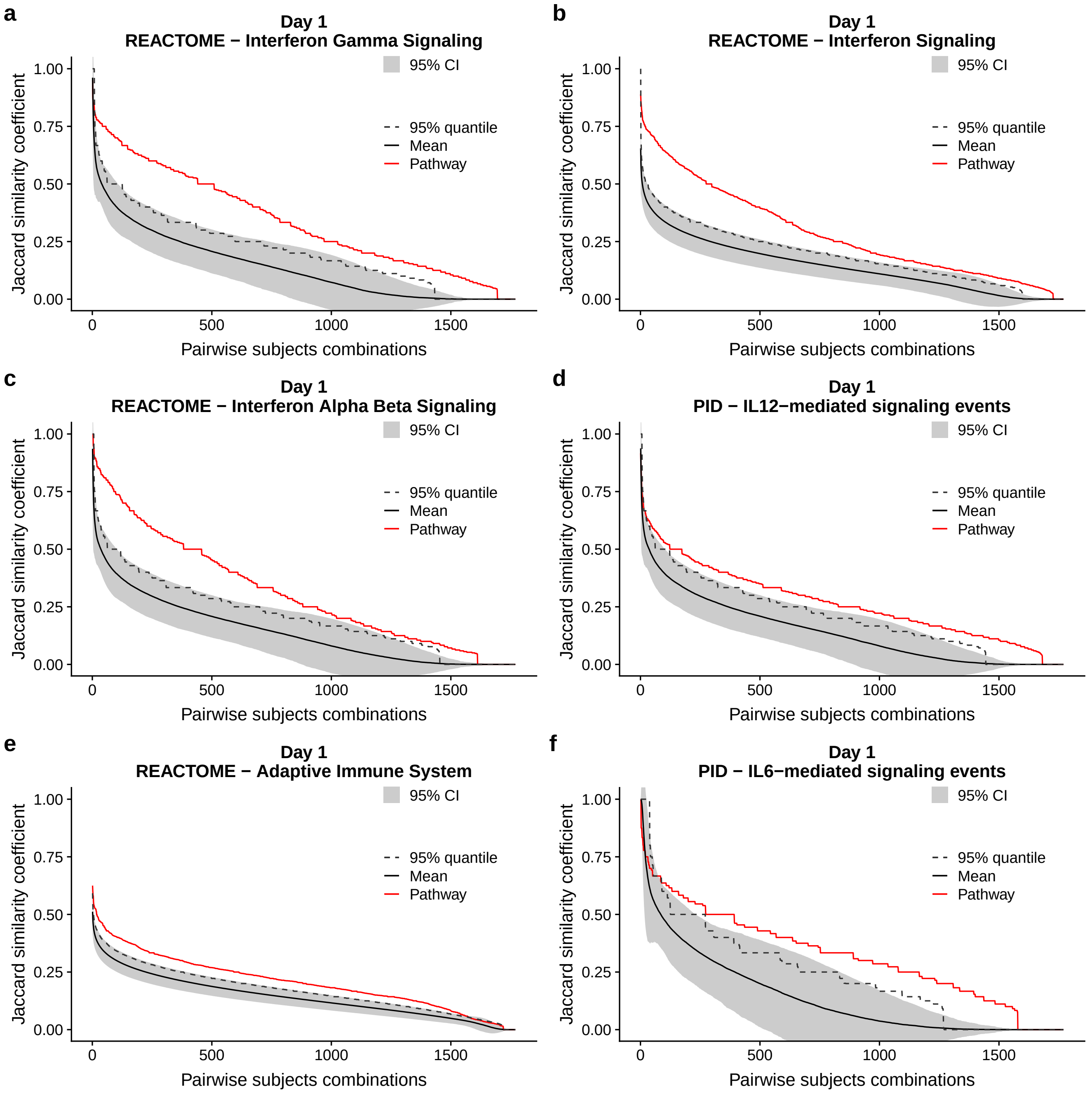

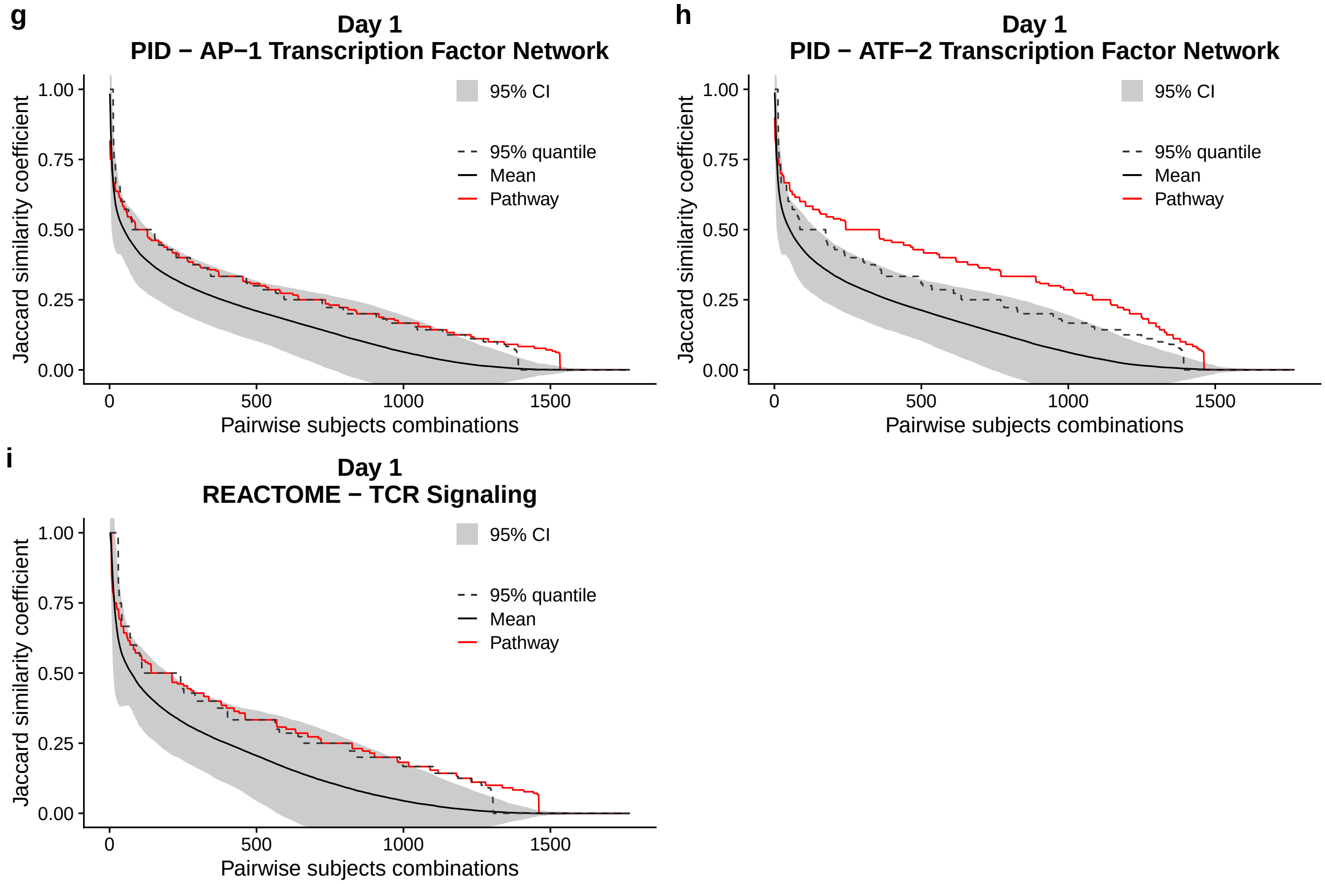

Figure S5. Most frequently enriched pathways at day 1. Jaccard similarity coefficients represent the robustness of gene modulation for those genes belonging to a specific pathway (red line). For comparison, the robustness of gene modulation of non-in-pathway, randomly selected genes (1000 bootstrap samplings) is also shown. Solid and dashed black lines represent the median and 95th percentile of the bootstrapped distributions. Gray shaded areas represent the 95% confidence interval.

Supplementary figure S6: Most frequently enriched pathways within day 7 individual subject transcriptomes (all subjects, top 10: 2 to 10)

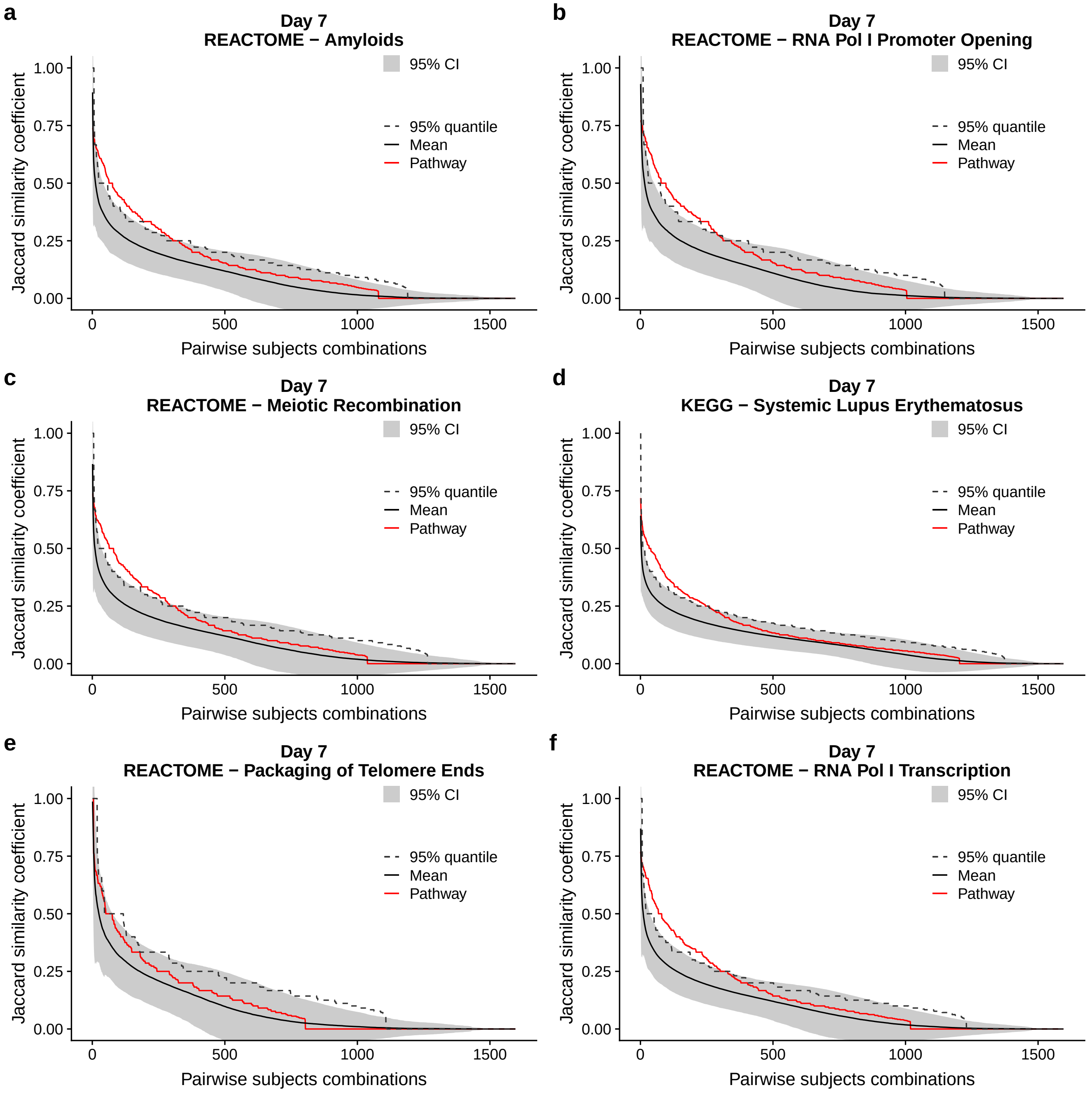

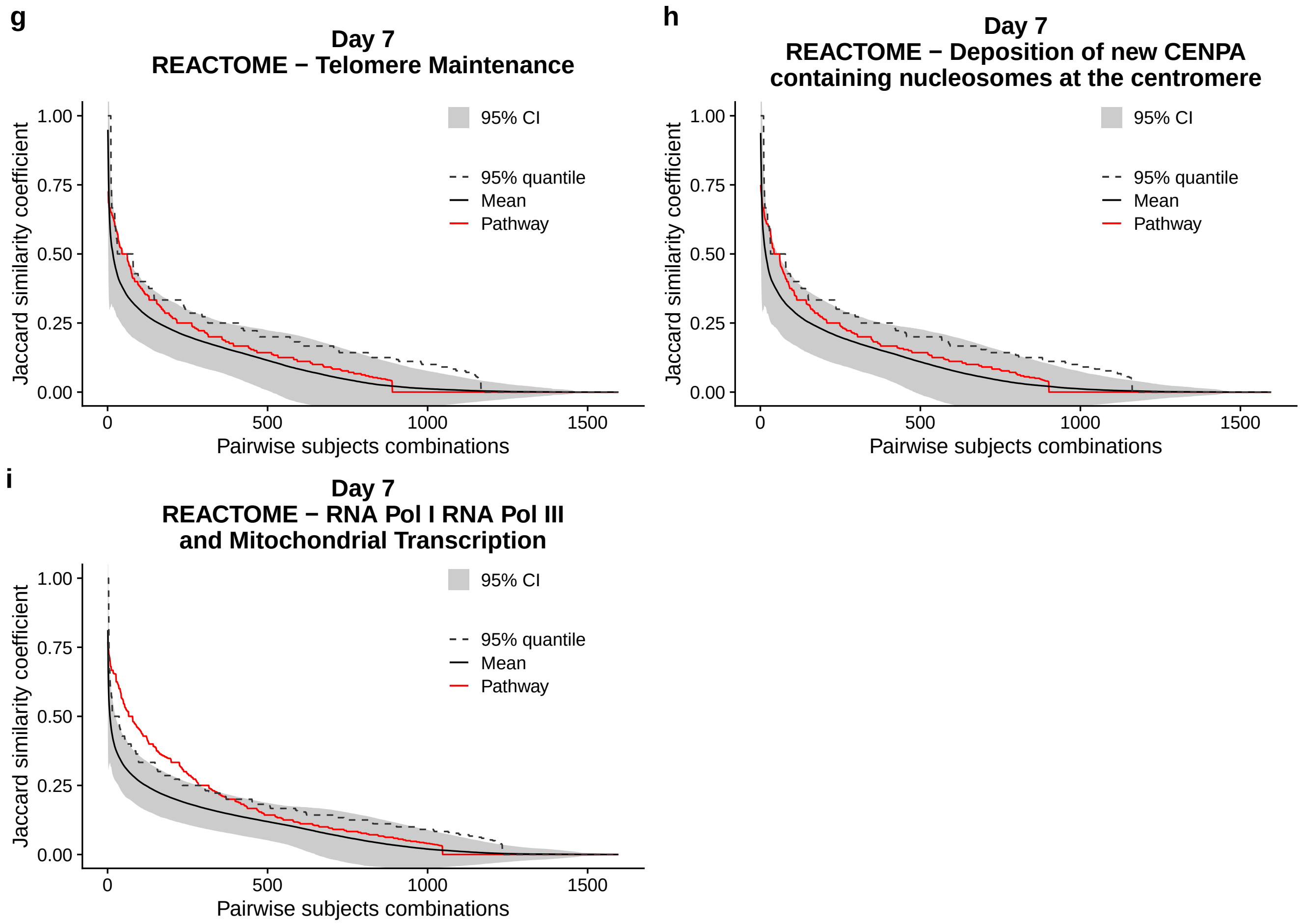

Figure S6. Most frequently enriched pathways at day 7. Jaccard similarity coefficients represent the robustness of gene modulation for those genes belonging to a specific pathway (red line). For comparison, the robustness of gene modulation of non-in-pathway, randomly selected genes (1000 bootstrap samplings) is also shown. Solid and dashed black lines represent the median and 95th percentile of the bootstrapped distributions. Gray shaded areas represent the 95% confidence interval.

Supplementary figure S7: Transcriptome response profiles for the day 1 vaccination-induced gene pool

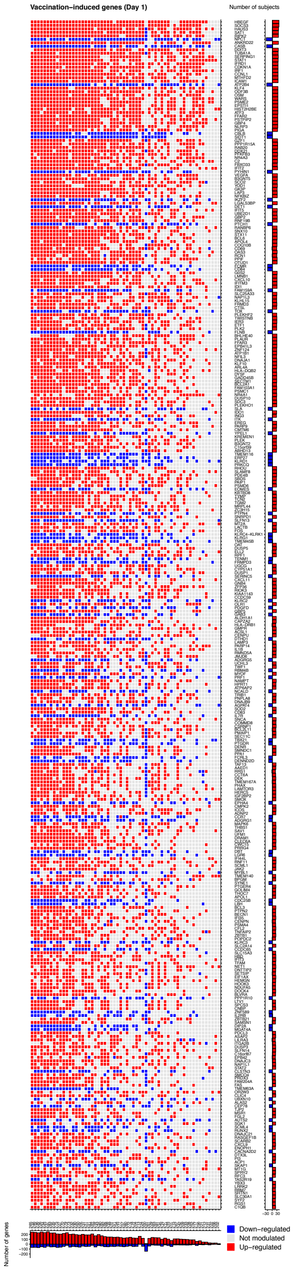

Figure S7. Individual transcriptome response profiles for the day 1 vaccination-induced gene pool. Each row corresponds to a gene and each column to a subject. On the right: number of gene modulations observed across all subjects. On the bottom: number of modulated genes in each study subject.

Supplementary figure S8: Percentages of vaccination-induced gene pool modulation for Day 1 data

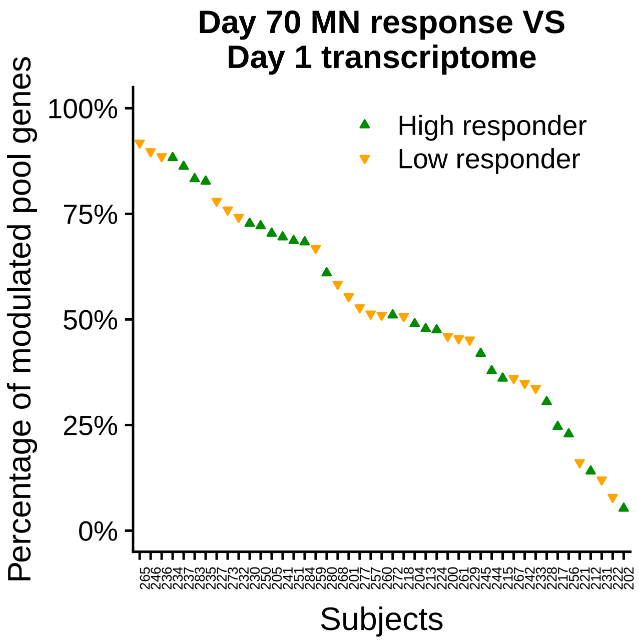

Figure S8. Percentage of modulation in the day 7 vaccination-induced gene pool shown in relation to the immunological response classification based on day 70 Influenza MicroNeutralization titers.

Supplementary Figure S9: Vaccine specific IgG-secreting cells (seasonal vaccine) vs transcriptome

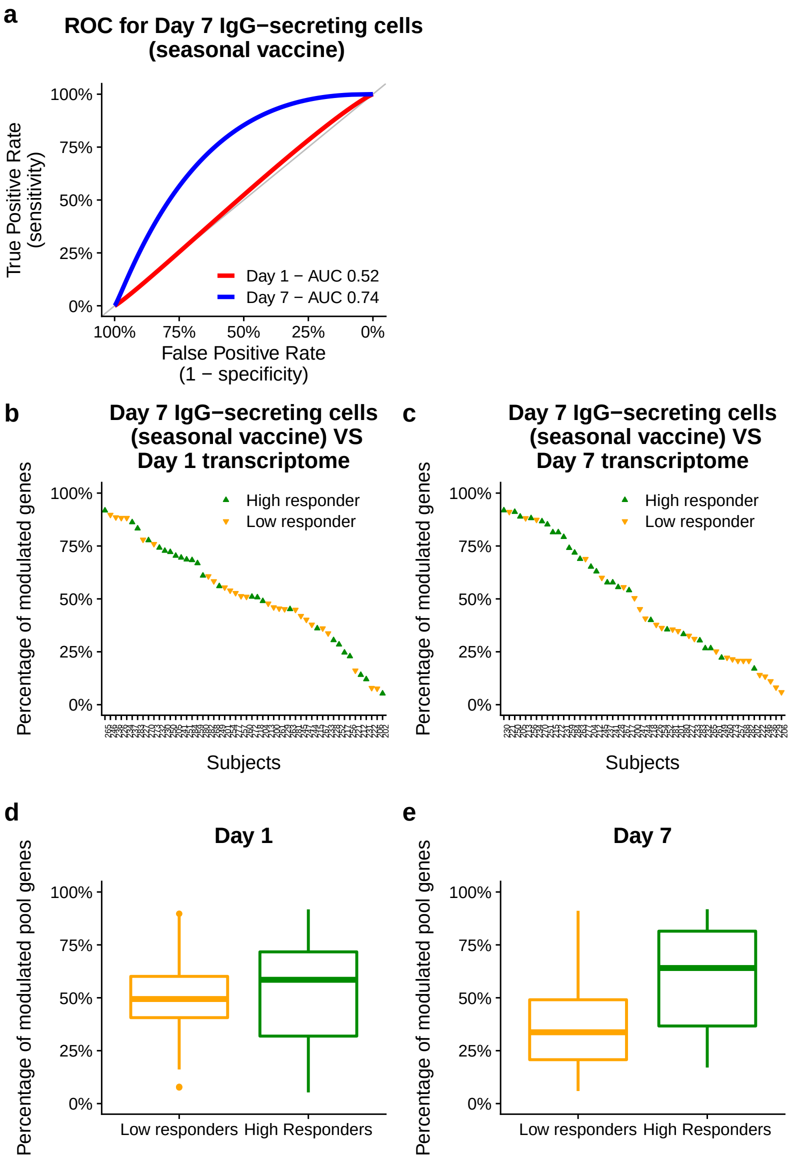

Figure S9. **a** Smoothed ROC curves of the immunological response classification predictions inferred using the modulation percentages of the vaccination-induced gene pools calculated from the individual transcriptome response profiles. **b-c** Percentage of modulation in the vaccination-induced gene pool shown in relation to the immunological response classification based on day 7 Influenza vaccine specific IgG-secreting cells. **d-e** Distribution of vaccination-induced gene pools modulation percentages grouped by immunological response classification.

Supplementary Figure S10: Vaccine specific IgG-secreting cells (H1N1 vaccine) vs transcriptome

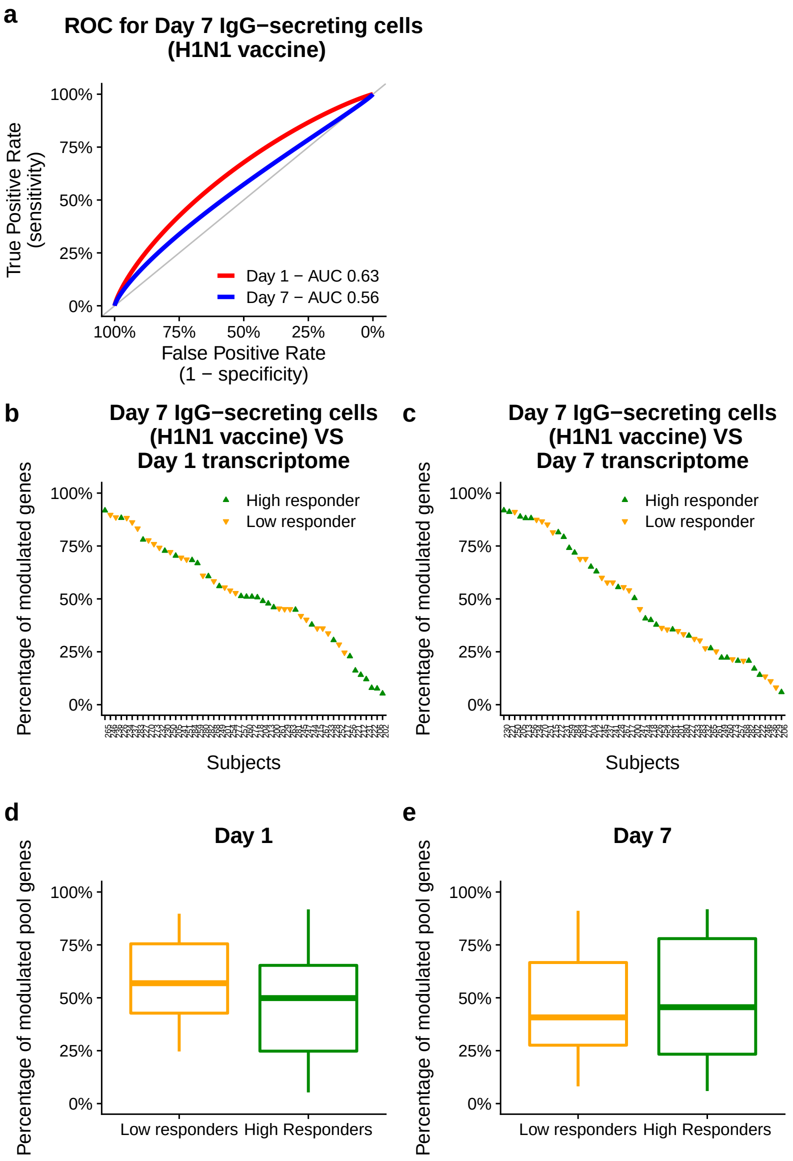

Figure S10. **a** Smoothed ROC curves of the immunological response classification predictions inferred using the modulation percentages of the vaccination-induced gene pools calculated from the individual transcriptome response profiles. **b-c** Percentage of modulation in the vaccination-induced gene pool shown in relation to the immunological response classification based on day 7 Influenza vaccine specific IgG-secreting cells. **d-e** Distribution of vaccination-induced gene pools modulation percentages grouped by immunological response classification.

Supplementary figure S11: Percentages of vaccination-induced gene pool modulation in relation to Day 0 MN titers

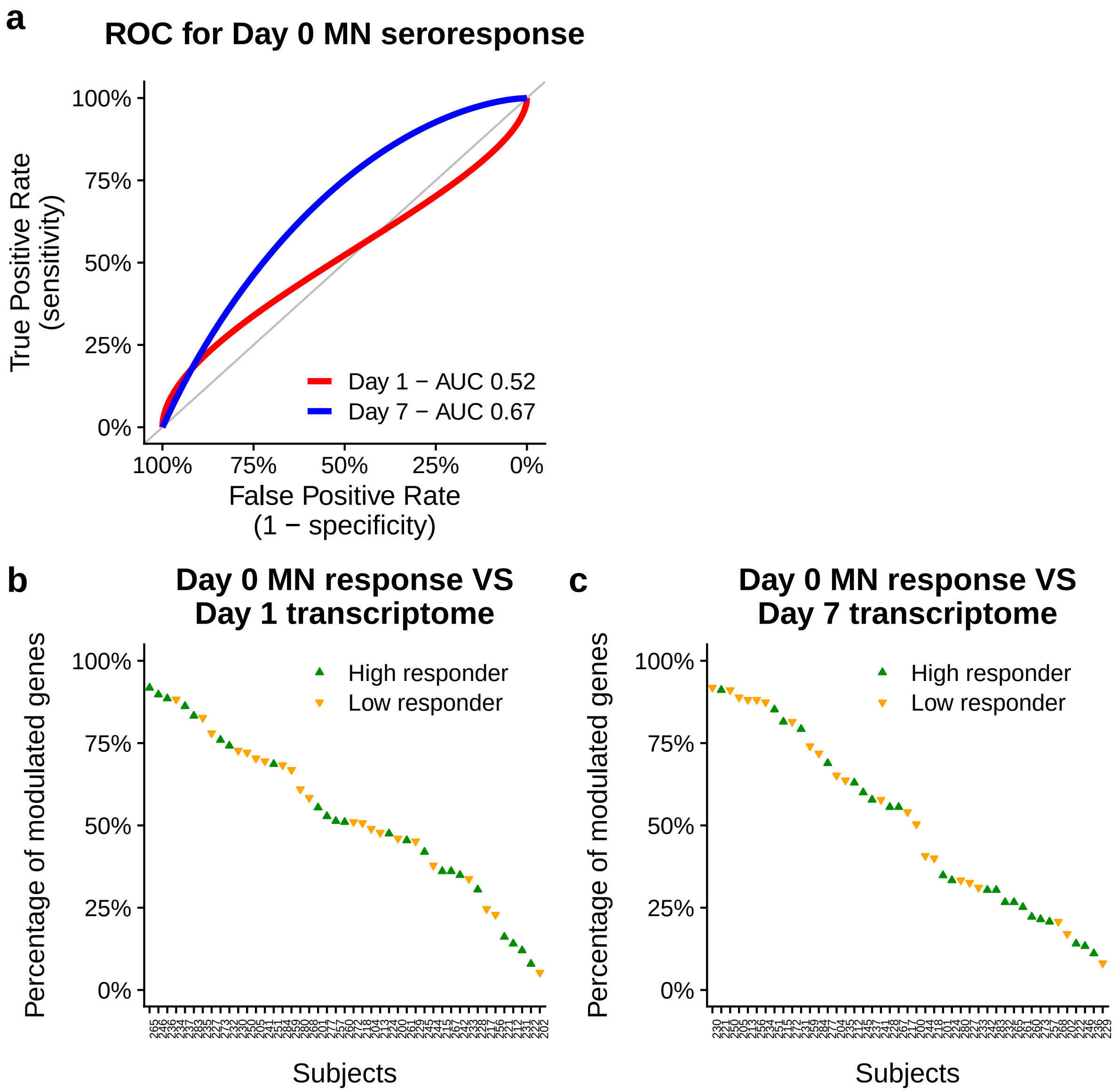

Figure S11. **a** Smoothed ROC curves of the immunological response classification predictions inferred using the modulation percentages of the vaccination-induced gene pools calculated from the individual transcriptome response profiles. **b-c** Percentage of modulation in the day 1 and day 7 vaccination-induced gene pool shown in relation to the immunological response classification based on day 0 Influenza MicroNeutralization titers.
